# Effects of drug treatments and types of drugs used by pregnant women at different gestational ages on pregnancy outcomes: A retrospective study

**DOI:** 10.1097/MD.0000000000041646

**Published:** 2025-03-14

**Authors:** Yuan Liu, Shaoneng Xiang, Yanying Wang, Qinghua Xu

**Affiliations:** a Department of Pharmacy, Jianyang Maternal and Child Health Hospital, Chengdu, China; b Department of Clinical Medicine, Jianyang Chinese Medicine Hospital, Chengdu, China.

**Keywords:** drug treatment, drugs, gestational age, pregnancy, pregnancy outcome

## Abstract

To analyze the effects of drug treatments and types of drugs on pregnancy outcomes in pregnant women at different gestational ages. The records of 526 pregnant women from our hospital from September 2018 to January 2024 were analyzed retrospectively. Women were categorized into 3 groups: normal delivery, artificial abortion, and spontaneous abortion. Data on maternal age, gestational age, smoking, drinking, radiation, medication timing, and types were collected and compared. Pearson correlation analysis assessed relationships between pregnancy outcomes, gestational age at medication, and medication types. After comparing multiple potential influencing factors, it was found that smoking history, timing of medication, and the use of antitussive and phlegm-reducing medications differed significantly among the different pregnancy outcome groups (*P* < .05). Multiple regression analysis showed that the gestational age at which pregnant women took medication was a significant positive influencing factor for adverse pregnancy outcomes, specifically artificial abortion (Coefficient = 0.210, *P* = .002). In addition, the use of antitussive and phlegm-reducing medications had a positive directional influence on adverse pregnancy outcomes, specifically spontaneous abortion (Coefficient = 0.294, *P* = .016). Further analysis showed that as the gestational age at the time of medication increased, the normal delivery rate initially increased and then decreased. The rate of artificial abortion first increased and then stabilized, while the spontaneous abortion rate showed minimal fluctuation. The use of medications by pregnant women and the duration of pregnancy significantly impact induced abortion rates. Cough and mucus-reducing medications can lead to miscarriage, while other drugs in early pregnancy generally do not affect outcomes. Antitussive and mucus-reducing drugs specifically increase the risk of spontaneous abortion. Raising awareness about proper medication use and conducting research on this topic is crucial for enhancing prenatal and postnatal care, reducing induced abortions, and promoting population growth.

## 1. Introduction

Medication use in pregnant women is a common clinical problem faced by health care professionals worldwide. Approximately 200 million women become pregnant every year, and ≈95% of pregnant women take >1 drug during pregnancy.^[1]^ However, most drugs lack safety data for use during pregnancy, primarily because clinical trials often exclude pregnant women. This poses significant challenges for health care providers when selecting medications for pregnant women. While some drugs are considered relatively safe for fetal development, concerns remain about the potential risks of many common medications. Currently, evidence on the effects of drug exposure during pregnancy in humans primarily comes from retrospective studies and postmarketing surveillance.^[2]^ For example, some studies have shown that over-the-counter emergency contraceptive pills do not significantly alter reproductive health outcomes,^[3]^ and a Swedish study found that exposure to allergic immunotherapy from 3 months before conception through the 22nd week of pregnancy does not increase the risk of congenital malformations.^[4]^ There is a statistically significant and clinically relevant association between sedative use during pregnancy and reduced birth weight.^[5]^ However, some medications, such as antiepileptic drugs, pose certain risks to fetal development.^[6]^ It remains challenging to protect newborns from the effects of drugs fully. Moreover, there is limited understanding of how different drugs impact the fetus during specific periods of pregnancy, necessitating further in-depth research. Generally speaking, the safety of medication for pregnant women is one of the critical issues that needs to be solved in the current medical field.

During pregnancy, physiological changes in maternal and placental-fetal units can affect the absorption, distribution, metabolism, and clearance of xenobiotic substances, including therapeutic and environmentally exposed substances.^[7]^ In addition, pregnancy may affect the levels and changing trends of multiple sex hormones in the body.^[8]^ Some studies have found that exposure to drugs such as domperidone and favipiravir in early pregnancy has fetal/neonatal malformation rates that are comparable to those in the unexposed group.^[9,10]^ However, other studies suggest that the risk of teratogenesis after drug use may be overestimated, and this overestimation could be more detrimental than the harm caused by inadequate drug knowledge.^[11,12]^ The lack of adequate consultation in community pharmacies increases the possibility of harmful reactions when pregnant women take medicines by themselves.^[13]^ Because pregnancy is prone to respiratory diseases and increases the tendency of mucosal edema, women may be more susceptible to acute sinusitis, thus increasing the risk of using related drugs.^[14]^ Prescription drug use among pregnant women continues to grow in developed countries such as Canada.^[15]^ Although antibiotic sales dropped from 2011 to 2018, only 6.25% of pharmacists asked about influencing factors such as allergic history and pregnancy status.^[16]^ Current safety assessments for medications used by pregnant women primarily focus on antiallergic drugs, antibiotics, antipsychotic drugs, and antiinfective drugs. However, there is a lack of survey data regarding the use of human papillomavirus vaccines and Coronavirus disease 2019 vaccines. For example, loratadine and some second-generation antihistamines have similar safety profiles in pregnant women.^[17]^ Penicillin has been used for a long time, has no noticeable harmful effects on the fetus, and remains the safest choice during pregnancy.^[18]^ Drugs may impact the development of cognitive and language skills later in life.^[19]^ Due to the lack of accurate information about the pregnancy cycle, it is challenging to assess medication use accurately. Medication use may also lead to increased ultrasound examinations and a higher likelihood of cesarean delivery.^[20]^ During pregnancy, physiological changes in the maternal and placental-fetal units influence xenobiotics’ absorption, distribution, metabolism, and excretion, including drugs prescribed for therapeutic purposes or chemicals to which women are inadvertently exposed from the surrounding environment. Optimizing maternal drug safety while reducing fetal harm requires a detailed understanding of drug safety in different pregnancy cycles.

We hypothesized that the effects of certain relatively safe drugs used before pregnancy, within 1 month of pregnancy, and during 2 to 3 months of pregnancy would vary in pregnancy outcomes. This study aimed to enhance the understanding of drug safety during human pregnancy, a significant public health issue worldwide. The results can guide evidence-based clinical practices to benefit mothers and their developing infants. By reviewing drug therapy in pregnant women at different gestational ages, we sought to determine if treatment responses and pregnancy outcomes differed based on drug exposure and its category. Identifying these effects could help optimize treatment strategies and counseling for pregnant women and inform improved prescribing guidelines to balance maternal and fetal health.

## 2. Materials and methods

### 2.1. Study design and ethics

This retrospective observational study was approved by the Ethics Committee of Jianyang Maternal and Child Health Hospital (Approval No. 2018-35). As per national regulations and institutional guidelines, written informed consent from participants was not required due to the study’s retrospective nature. The study adhered to the principles outlined in the Declaration of Helsinki.

### 2.2. Baseline data

The medical records and data of 526 pregnant women who visited our hospital from September 2018 to January 2024 were retrospectively analyzed and categorized into 3 groups based on pregnancy outcomes: normal delivery, artificial abortion, and spontaneous abortion. Inclusion criteria were: pregnant women who visited the obstetrics department of our hospital from September 2018 to January 2024 and took medication during the first trimester or early pregnancy; complete clinical data records, including basic information, initial pregnancy diagnosis, medication use, and pregnancy outcomes; and clear documentation of gestational age at medication, categorized as before the last menstrual period, 1 to 2 weeks after the last menstrual period, or 3 to 8 weeks after the last menstrual period. Exclusion criteria were: cases with significant missing data preventing determination of key variables such as pregnancy timing and medication status; cases of pregnancy termination due to miscarriage or other early pregnancy issues; cases with high-risk factors such as fetal anomalies; cases where the type of medication used is unclear or involves long-term drug treatment; and cases where the pregnancy outcome could not be determined. A normal delivery is defined as the delivery of a normal fetus. Spontaneous abortion includes cases of fetal malformation, stillbirth, or artificial abortion with unclear reasons, while artificial abortion is classified as cases with no clear reason. The final study cohort included 345 cases in the normal delivery group, 166 cases in the artificial abortion group, and 14 cases in the spontaneous abortion group.

### 2.3. Data collection method

Basic information extracted using a general information questionnaire includes age, parity, smoking history, drinking history, radiation history, and gestational age at medication. Medication usage data were collected from the consultation entry system and medical records, focusing on medication types such as antiinfective drugs, antiinflammatory and analgesic drugs, cold and rhinitis medications, nutritional supplements, probiotics, Chinese patent medicines, Coronavirus disease 2019 vaccines, and external gynecological drugs. Pregnancy outcomes were clarified through telephone calls or follow-up visits, including delivery conditions (e.g., normal delivery, early premature birth) and neonatal status (e.g., induction of labor or artificial abortion). Detailed information about medication types and names is presented in Table 1.

**Table 1:** Corresponding detailed names of the types of drugs used in this study.

Type of medication	Specific name of the medication
Antibiotics drugs	Cefixime, amoxicillin, levofloxacin, cefprozil, tinidazole, norfloxacin, etc
Antiviral drug	Acyclovir, valacyclovir, ribavirin, oseltamivir, etc
Antipyretic analgesics	Ibuprofen, compound phenamine granules
Hormonal	Prednisone, dexamethasone, prednisone, etc
Nutritional supplements	vitamin B1 tablets, vitamin C, etc
Vaccines	New Coronavirus adenovirus nucleic acid vaccine
Antiallergic drug	loratadine tablets, chlorpheniramine maleate tablets, and Levocetirizine
Oral contraceptives	Levonorgestrel
Digestive system	Omeprazole
Cough and phlegm medicines	Compound licorice tablets, Kebiqing, Eucalyptol, Limonene and, Pinene Enteric Soft Capsules, etc
Antimicrobial	Albendazole
Anesthetics	2% lidocaine injection, 4% articaine, bupivacaine
Chinese patent medicine/decoction	Tongmai Granules, Rupi Sanjie Capsules, etc

### 2.4. Statistical methods

Data analysis was performed using SPSS 26.0 (https://www.ibm.com/support/pages/spss-statistics-260-fix-pack-1) statistical software. For data that met the normal distribution and had homogeneous variances, the 2-sample independent *t* test was used for group comparisons. Nonnormally distributed measurement data were described using median (Q1–Q3), and the nonparametric rank sum test was applied. Enumeration data were described by case counts, and the χ² test was used for analysis. Influencing factors were determined using binary logistic regression analysis; correlation analysis was performed using Pearson correlation analysis. The correction level is α = 0.05, and *P* < .05 is regarded as a statistically significant difference. The visual analysis uses Origin 2021 software to draw histograms and line charts.

## 3. Results

### 3.1. Comparing the general information of the 3 groups

A comparative analysis was conducted on the baseline characteristics of women with different pregnancy outcomes: normal delivery (n = 345), induced abortion (n = 167), and spontaneous abortion (n = 14). The median age was slightly higher in the induced and spontaneous abortion groups (28 and 28.5 years, respectively) compared to the normal delivery group (27 years). However, this difference was not statistically significant (*P* = .263). There was no statistically significant difference in age, parity, smoking history, drinking history, and radiation history among the 526 pregnant women included in this study (*P* > .05). The results revealed significant differences in smoking history (*P* = .003), timing of medication during gestation (*P* = .040), and the use of cough and phlegm medicines (*P* = .000) across the groups (Table 2).

**Table 2:** Compare baseline data of women with different pregnancy outcomes.

Equation	Normal delivery (n = 345)	Induced abortion (n = 167)	Spontaneous abortion (n = 14)	χ^2^/*t*	*P*
Age (yr)	27 (25–30)	28 (25–32)	28.5 (26–35)	2.661	.263
Smoking history (cases)	2	0	1	11.595	.003
Drinking history (cases)	9	8	0	2.229	.328
Radiation history (cases)	5	2	0	0.245	.885
Medication gestational age. (cases)				10.033	.040
Fertilization	25	3	3		
1 to 2 wk before fertilization	182	78	12		
3 to 8 wk before fertilization.	138	85	7		
Medication types (cases)					
Antibiotics drug	179	90	12	0.532	.767
Antivirals	16	14	1	1.501	.472
Antipyretic analgesics	84	37	1	2.163	.339
Hormones	23	16	1	0.832	.660
Nutritional supplements	22	15	1	0.603	.740
Vaccines	44	13	2	1.588	.452
Antiallergy drugs	18	16	0	1.207	.547
Oral contraceptives	13	13	1	1.365	.505
Digestive system	22	16	0	2.585	.275
Anaesthetics	12	1	1	4.301	.116
Antimicrobial	1	1	0	0.344	.842
Cough and phlegm medicines	35	15	7	15.496	.000
Chinese patent medicine/decoction.	218	118	20	2.432	.300

### 3.2. Distribution of medicine types used by women

**Figure 1.**
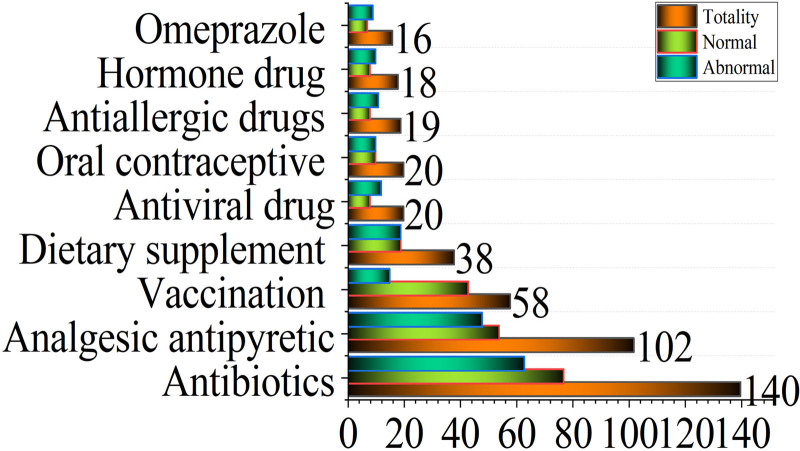
Type of drug use in pregnant women. The data in the figure represents the total number of application cases; normal delivery refers to natural delivery; induced abortion refers to artificial abortion; spontaneous abortion refers to spontaneous abortion. Numbers represent the number of cases of spontaneous delivery of medication.

The distribution of types of medicines used by women shows that antibiotics have the highest usage (31.56%), followed by analgesic antipyretics, while anesthetics and antiparasitic drugs are rarely used (Fig. 1).

### 3.3. Multiple regression analysis of adverse pregnancy outcomes

According to the assignment method (natural delivery = 0, spontaneous abortion = 1, and induced abortion = 2), smoking history, use of antitussive and phlegm drugs, and gestational age were included in the multiple regression analysis. It was found that the gestational age of pregnant women using drugs was a significant positive predictor of adverse pregnancy outcomes (Coefficient = 0.210, *P* = .002). Smoking history and the use of antitussive and phlegm medications were not significant predictors (*P* > .05) (Table 3). In addition, multiple regression analysis was performed using assignment methods: natural delivery = 0, artificial abortion = 1, and spontaneous abortion = 2. It was found that smoking history and the gestational age at which medication was administered did not affect the pregnancy outcome. However, the use of antitussive and phlegm medication was associated with adverse pregnancy outcomes, producing a positive impact (Coefficient = 0.294, *P* = .016) (Table 4).

**Table 3:** Risk factors for adverse pregnancy outcomes (induced abortion).

Independent variables	Coefficient	SE	*t*	*P*	*r*_partial_	*r*_semipartial_	VIF
Constant	0.164						
Medication gestational age	0.210	0.039	3.060	.002	0.133	0.133	1.008
Smoking history	−0.460	0.535	−0.860	.390	-0.038	0.037	1.007
Cough and phlegm medicines	−0.038	0.179	−0.214	.830	-0.009	0.009	1.002

SE = standard error, VIF = variance inflation factor.

**Table 4:** Risk factors for adverse pregnancy outcomes (spontaneous abortion).

Independent variables	Coefficient	SE	*t*	*P*	*r*_partial_	*r*_semipartial_	VIF
Constant	0.168						
Medication gestational age	0.568	0.364	1.560	.119	0.068	0.068	1.007
Smoking history	0.088	0.047	1.888	.060	0.082	0.082	1.008
Cough and phlegm medicines	0.294	0.122	2.413	.016	0.105	0.105	1.002

SE = standard error, VIF = variance inflation factor.

### 3.4. Pregnancy outcomes of women taking medication at different gestational ages

**Figure 2.**
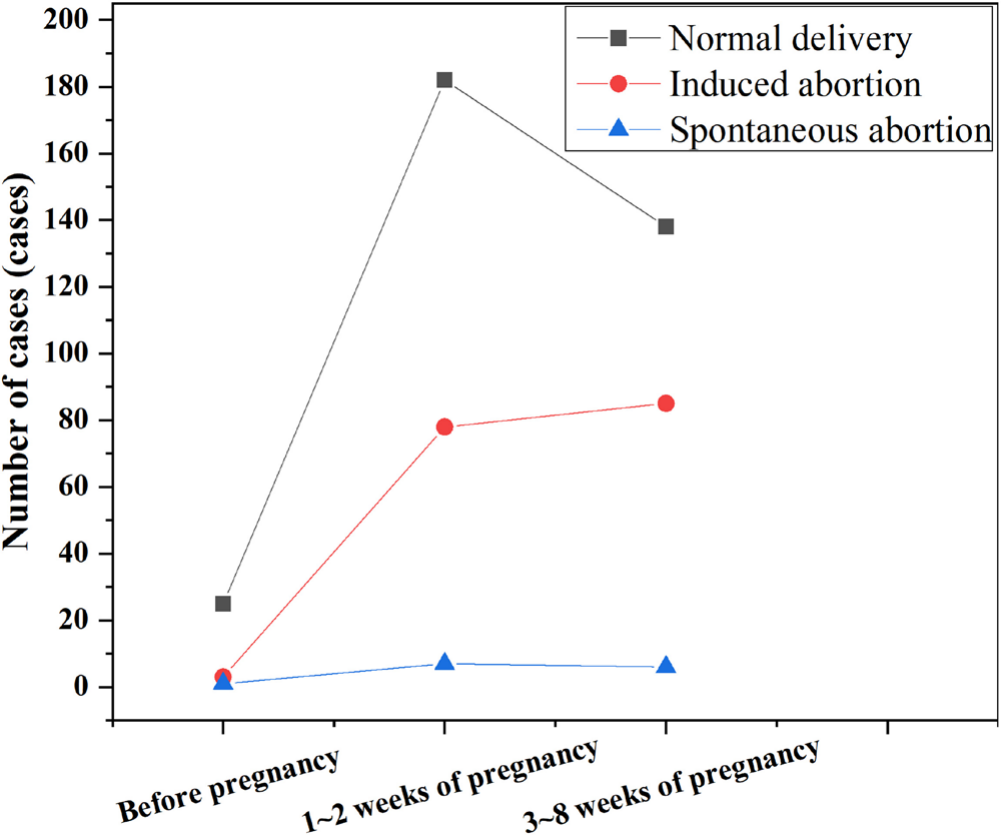
Trends in pregnancy outcomes of medication use at different gestational ages. The abscissa in the figure is the gestational age of medication, the ordinate is the number of pregnant women, and the lines represent the changing trend.

The incidence rate of spontaneous abortion among 29 pregnant women with a history of drug use before pregnancy was 3.45% (1/29), while the incidence rate of artificial abortion was 10.34% (3/29). Among 267 pregnant women with a history of drug use during 1 to 2 weeks of pregnancy, the incidence rate of spontaneous abortion was 2.62% (7/267), and the incidence rate of artificial abortion was 29.21% (78/267). For 229 pregnant women taking medication between 3 and 8 weeks of gestation, the incidence rate of spontaneous abortion was 2.62% (6/229), and the incidence rate of artificial abortion was 3.06% (7/229). The overall incidence rate was 37.12% (85/229), and the difference between the groups was statistically significant (χ^2^ = 10.033, *P* < .001). As the gestational age at the time of medication increased, the number of pregnant women with normal deliveries initially increased and then decreased. Pregnant women with artificial abortions showed a trend of initial increase followed by stabilization, while the fluctuation in the number of pregnant women with spontaneous abortions remained minimal, as shown in Figure 2.

### 3.5. The relationship between the gestational age at the time of medication, the type of medication, and pregnancy outcomes

Our results indicated that there is a positive correlation between gestational age and adverse pregnancy outcomes (*r* = 0.091, *P* < .05). In addition, there is a positive correlation between cough and expectorant drugs and adverse pregnancy outcomes (*r* = 0.106, *P* < .05; Table 5).

**Table 5:** Relationship between gestational age and types of medication and pregnancy outcomes.

Equation	Pregnancy outcome	
	*r*	*P*
Medication gestational age	0.091	.037
Antibiotics drug	0.014	.741
Antivirals	−0.004	.934
Antipyretic analgesics	0.023	.605
Hormones	0.001	.981
Nutritional supplements	0.033	.455
Vaccines	−0.051	.247
Antiallergy drugs	−0.004	.924
Oral contraceptives	0.042	.339
Digestive system	0.009	.841
Anaesthetics	0.032	.461
Antimicrobial	0.010	.816
Cough and phlegm medicines	0.106	.015
Chinese patent medicine/decoction	0.030	.496

## 4. Discussion

Pregnancy is a unique time in every woman’s life.^[21]^ This study preliminarily found that medication use at different gestational ages affects pregnancy outcomes, with medication use at later gestational ages increasing the risk of induced abortion. These findings are consistent with results reported in previous studies.^[22]^ The embryonic period from 3 to 8 weeks after fertilization is considered the “teratogenic highly sensitive period,” during which the development of various fetal systems is particularly susceptible to the effects of drugs. Pregnant women are also more concerned about the impact of medication, which may increase the likelihood of choosing induced abortion.^[23]^ While this study has identified that the use of antitussive and phlegm-reducing drugs increases the risk of spontaneous abortion, it remains uncertain whether many medications in early pregnancy elevate the risk of adverse outcomes. However, previous studies examining the effects of cough medicine, such as dextromethorphan, on pregnancy outcomes have not documented an increased risk of major malformations.^[24,25]^ Medical staff should address knowledge gaps regarding the specific characteristics of pregnant women at different gestational ages and the potential risks of various drugs.

From the baseline data of the 526 pregnant women included in this study, their age, drinking history, and radiation history are all balanced, indicating that the data are relatively reliable. However, most studies examine the impact of different gestational ages on early pregnancy outcomes. It is suggested that clinical safety supervision of drug treatment for pregnant women should be strengthened, particularly during the early stages of pregnancy, especially the second and third months. Medications should be used with caution during this period.^[26]^ Antibiotics were most frequently used in terms of drug types, followed by analgesics and antipyretics. Anesthetics and antiparasitic drugs were rarely prescribed. Given the increased susceptibility of pregnant women to infections and to assess the impact of these drug types on pregnancy outcomes, the study indicates that the appropriate use of antibiotics, antipyretics, and analgesics is generally safe for pregnant women. However, infections during pregnancy can increase the risk of antibiotic use.^[27,28]^ Antibiotic exposure during pregnancy can have systemic effects on fetal and maternal health, primarily impacting glucose metabolism.^[29,30]^ A recent meta-analysis^[31]^ assessing the prevalence of antibiotic use during pregnancy observed a range of 0.04% to 90%, with a pooled prevalence estimate of 23.6%, while in this study, the rate was ≈31.56%. In line with our findings, a study conducted in Saudi Arabia found that 26.4% of pregnant women received antibiotic prescriptions, with most of these prescriptions given to women over 30 years old.^[32]^

Antibiotic use during pregnancy differs markedly between regions, with notably higher rates in the Western Pacific and the Americas.^[31]^ This variation is influenced by overprescription, self-medication, disease prevalence, socioeconomic conditions, and environmental factors. Addressing these differences is essential for designing targeted interventions to minimize unnecessary antibiotic use and enhance maternal and fetal health. Additionally, gestational age and the timing of medication use are independent risk factors affecting pregnancy outcomes, providing a reliable basis for clinical practice. The odds ratio is 2.384 for the gestational age at which the drug is taken, indicating that the risk increases by ≈2.4 times. This underscores the importance of paying attention to the timing of drug use during pregnancy. In addition, increasing maternal age elevates the risk, and even if the impact is limited, the hormonal changes in older pregnant women should be considered. The gestational age at which medication is used is positively correlated with outcomes, suggesting that later drug use should be minimized. Specifically, the later the gestational age at which medication is used (Coefficient = 0.210, *P* = .002), the higher the risk of induced abortion as an outcome. In this study, the use of antitussive and phlegm-reducing drugs is linked to an increased risk of spontaneous abortion (Coefficient = 0.294, *P* = .016). Nonetheless, previous studies have not demonstrated a significant association between dextromethorphan and an elevated risk of congenital disabilities during pregnancy.^[33,34]^ In our study, most patients utilized cough medications, including compound Licorice Tablets, Kebiqing, Eucalyptol, Limonene, and Pinene Enteric Soft Capsules. However, there is currently insufficient scientific evidence to confirm the safety of some of these medicines, such as Kebiqing, for pregnant women and their fetuses.^[35]^ Given the absence of definitive safety data, it is prudent to exercise caution with its use. Carefully weighing the potential risks and benefits and consulting with a health care provider is crucial when considering any medication during pregnancy. Prior studies have found that young age, unintended pregnancy, being in the first week of early pregnancy, a history of high-risk pregnancies, and failure to receive routine prenatal care are independent variables for predicting moderate to severe anxiety.^[36]^ It is evident that factors such as age and unintended pregnancy are related to pregnancy outcomes. However, the results of this study are consistent with most existing studies, which suggest that the gestational age of medication use is linked to pregnancy outcomes; this finding has been partially verified.

This study has several limitations. First, the relatively small sample size limits generalizability, and the research was conducted at a single center, which may not fully capture variability across different settings. Second, the study lacks detailed baseline characteristics and comprehensive drug use information, such as disease type and dosage. In addition, the absence of classification for different types of drugs could impact the findings. Third, regional differences in antibiotic use standards may further affect the results. Future research should address these limitations through larger, multicenter studies with detailed data and long-term follow-up to validate and extend these findings.

### 4.1. Implications for healthcare and policymakers

The study underscores the need for updated clinical guidelines on medication use during pregnancy, emphasizing caution, especially in early gestation. Healthcare providers should adhere to new safety protocols and consider gestational age when prescribing medications to mitigate risks. Policymakers should support the development of more stringent drug safety regulations and advocate for comprehensive research to validate these findings through large-scale, multicenter studies. In addition, personalized medication strategies based on individual risk factors should be promoted to enhance safety. Implementing systematic medication tracking and management models will help monitor and adjust medication use effectively. Public health initiatives should focus on educating health care providers and pregnant women about medication risks and safety measures, ensuring informed decision-making and improved health outcomes.

## 5. Conclusion

The study found that the use of certain medications, particularly antitussive and phlegm-reducing drugs, increases the risk of fetal dysplasia and miscarriage. This highlights the urgent need for improved awareness and education on medication effects during pregnancy. The absence of a government-level pregnancy medication registration system in China underscores the necessity for more robust guidelines and personalized medication services. Enhancing education on safe and rational medication use will empower pregnant women to make informed decisions, which is crucial for optimizing prenatal care and supporting population growth. Further research into drug safety and effects is essential for refining pregnancy care practices.

## Author contributions

**Conceptualization:** Yuan Liu, Shaoneng Xiang, Yanying Wang, Qinghua Xu.

**Data curation:** Yuan Liu, Yanying Wang, Qinghua Xu.

**Formal analysis:** Yuan Liu, Shaoneng Xiang, Yanying Wang, Qinghua Xu.

**Funding acquisition:** Yuan Liu, Shaoneng Xiang.

**Investigation:** Yuan Liu, Yanying Wang, Qinghua Xu.

**Methodology:** Yuan Liu, Shaoneng Xiang, Yanying Wang.

**Project administration:** Yuan Liu, Yanying Wang.

**Resources:** Yuan Liu, Shaoneng Xiang, Yanying Wang, Qinghua Xu.

**Supervision:** Yuan Liu, Qinghua Xu.

**Validation:** Yuan Liu, Shaoneng Xiang, Qinghua Xu.

**Visualization:** Yuan Liu, Shaoneng Xiang, Yanying Wang, Qinghua Xu.

**Writing—original draft:** Yuan Liu, Shaoneng Xiang, Yanying Wang, Qinghua Xu.

**Writing—review & editing:** Yuan Liu, Shaoneng Xiang, Yanying Wang, Qinghua Xu.

**Software:** Shaoneng Xiang.
